# Interlimb kinetic asymmetries during the tuck jump assessment are more exposed following kinetic stabilization

**DOI:** 10.1016/j.ptsp.2024.03.002

**Published:** 2024-03-18

**Authors:** Lucy S. Kember, Gregory D. Myer, Rhodri S. Lloyd

**Affiliations:** a School of Sport and Health Sciences, Cardiff Metropolitan University, Cardiff, UK; b Sport Performance Research Institute, New Zealand, Auckland University of Technology, Auckland, New Zealand; c Centre for Sport Science and Human Performance, Waikato Institute of Technology, Hamilton, New Zealand; d Emory Sport Performance and Research Center, Flowery Branch, GA, USA; e Emory Sports Medicine Center, Atlanta, GA, USA; f Department of Orthopaedics, Emory University School of Medicine, Atlanta, GA, USA; g The Micheli Center for Sports Injury Prevention, Waltham, MA, USA

**Keywords:** Repeated jumping, Asymmetry, Statistical parametric mapping, ACL

## Abstract

**Objective:**

To analyse interlimb kinetics and asymmetries during the tuck jump assessment (TJA), before and after kinetic stabilization, to identify injury risk in healthy female athletes.

**Design:**

Cross-sectional study.

**Setting:**

Laboratory.

**Participants:**

Twenty-five healthy females (age 21.0 ± 1.83 yrs; height 1.68 ± 0.06 m; body mass 69.4 ± 10.7 kg).

**Main outcome measures:**

Kinetics were measured during 10-s trials of the TJA and absolute asymmetries compared, before and after kinetic stabilization using paired sample t-tests. Statistical parametric mapping (SPM) compared vertical ground reaction force (VGRF) data for each limb during the jumping cycles before and after stabilization.

**Results:**

Small to moderate increases in interlimb asymmetries were observed after stabilization for VGRF, relative vertical leg stiffness, average loading rate, total and propulsive impulse, peak braking and propulsive force (*p* < 0.05). SPM revealed significant interlimb differences between 77–98% and 83–99% of ground contact for the jumping cycles pre- and post-stabilization respectively.

**Conclusions:**

Larger asymmetries were evident after kinetic stabilization, with increased VGRF in the non-dominant limb. We speculate that participants sacrificed interlimb landing symmetry to achieve kinetic stability, which may reflect a primal landing strategy that forgoes movement quality. Assessing lower limb biomechanics using the TJA should involve examining kinetic stability and interlimb kinetic asymmetries.

## 1. Introduction

Interlimb asymmetries, characterised by differences in movement patterns and performance between limbs, play a critical role in overall movement quality (Bishop, Turner, & Read, 2018a; Martonick et al., 2023). The presence of interlimb differences during various athletic tasks has been postulated to be a precursor to non-contact ACL injuries (Pappas & Carpes, 2012; Paterno, Ford, Myer, Heyl, & Hewett, 2007), and kinetic asymmetries exceeding 10% often persist in males and females 8–12 months post ACL reconstruction (ACLR) (Ardern, Webster, Taylor, & Feller, 2011; Hughes, Musco, Caine, & Howe, 2020). Vertical ground reaction forces (VGRF) have often been examined to assess interlimb kinetic asymmetries, specifically during single repetition bilateral jump-landing tasks such as a drop jump (Bakal et al., 2022a) and countermovement jump (Costley, Miles, King, & Daniels, 2023). However, it remains unclear whether interlimb kinetic asymmetries manifest during repeated jump-landing tasks, and the extent of their magnitude.

The tuck jump assessment (TJA) is a clinical screening tool used to visually assess landing movement patterns and deficits associated with lower limb injury (Fort-Vanmeerhaeghe, Montalvo, Lloyd, Read, & Myer, 2017; Herrington, Myer, & Munro, 2013; Myer, Ford, & Hewett, 2008). The assessment requires athletes to perform repeated tuck jumps over a 10-s period and requires adequate multijoint coordination and stretch-shortening cycle capabilities to perform the technical requirements of the task (Kember, Lloyd, Myer, & Moore, 2022; Read, Oliver, De Ste Croix, Myer, & Lloyd, 2016). The dynamic nature of the TJA presents a unique opportunity to observe how interlimb kinetics and asymmetries present during repeated jumping compared to a single jump-landing task. More specifically, rebounding tasks may increase the likelihood of an unstable body posture in-flight which could lead to greater landing perturbation and heightened interlimb asymmetries, especially during the initial jumping cycles as an athlete tries to find a consistent rebounding strategy (Kember et al., 2022). VanZile et al. (2021) recently investigated interlimb kinetic asymmetries during the first five cycles of repeated tuck jumps in ACLR patients. They found that kinetics were greater in the uninvolved limb compared to the involved limb, with asymmetries ranging from 15 to 35% for impact forces, 4–21% for loading rates, and 6–19% for propulsive forces (VanZile et al., 2021). Whilst such findings are novel in the assessment of ACLR asymmetry, and may indicate inadequate rehabilitation, the presence and magnitude of asymmetries in a non-clinical population are unknown and of particular interest in this study.

In healthy female athletes, bilateral kinetic analysis of the TJA and the assessment of kinetic stabilization has previously been explored (Kember et al., 2022). Kinetic stabilization refers to the point during a movement when an athlete attains a level of stability in their force-time profile through the coordination of various muscle groups and joint interactions (James, Herman, Dufek, & Bates, 2007). On average, 15 jumping cycles are performed by healthy females during a single trial of the TJA, with a minimum of 11 jumping cycles necessary to achieve kinetic stabilization across a range of discrete VGRF variables (Kember et al., 2022). The authors proposed that the assessment of movement deficits, including interlimb asymmetry, should not be assessed during a repeated tuck jump task until kinetic stabilization is achieved (Kember et al., 2022). Currently, the existing literature is limited to bilateral biomechanical assessment (Kember et al., 2022), therefore the relative contribution and variance in interlimb kinetics before and after kinetic stabilization during the TJA remain unknown.

Notwithstanding the limited evidence examining the kinetics of the TJA, existing studies have typically relied on reporting discrete, instantaneous data (e.g., peak force). More recently, the assessment of continuous time-series data has been employed using waveform analysis through statistical parametric mapping (SPM). This form of analysis has identified significant differences in interlimb kinetics during a variety of jumping tasks (Hughes-Oliver, Harrison, Williams, & Queen, 2019; Martonick, Chun, Krumpl, & Bailey, 2022; Martonick et al., 2023), and may provide more granular information to better inform clinicians and researchers when developing screening and rehabilitation interventions (Hogg et al., 2020; Hughes-Oliver et al., 2019; King et al., 2018). Waveform analysis of repeated tuck jump cycles, specifically before and after kinetic stabilization, could uncover between-limb asymmetries that may not be apparent through analysis of discrete variables alone.

Given the existing literature, this study aimed to provide detailed insight into the asymmetries of TJA kinetics, with the novel data being of use to the future assessment of injury risk and rehabilitation. Specifically, the study aimed to: (i) examine differences in interlimb kinetic asymmetries of discrete variables during the TJA before and after kinetic stabilization, and (ii) identify differences between limbs before and after kinetic stabilization using discrete time point and SPM waveform analysis. Our initial hypothesis was that participants would demonstrate greater interlimb asymmetries prior to kinetic stabilization, reflective of altered interlimb VGRF loading strategies.

## 2. Methods

### 2.1. Participants

Twenty-five female athletes (age 21.0 ± 1.83 yrs; height 1.68 ± 0.06 m; body mass 69.4 ± 10.7 kg) were recruited from the local university to take part in this study. A priori power analysis was conducted using G*Power (v.3.1.9.6) to test the difference between dependent means with a moderate effect size (ES) (*d* = 0.60) and an alpha of 0.05; results indicated that a total sample of *n* = 24 was required to achieve a power of 0.80 (Faul, Erdfelder, Lang, & Buchner, 2007). Inclusion criteria consisted of current participation in team sports (football n = 3, netball n = 20, and basketball n = 2); training age of at least three years (Myer, Lloyd, Brent, & Faigenbaum, 2013); and playing their sport at least three times per week. Participants were excluded if they had any diagnosed lower limb neuromuscular injuries within three months prior to testing. Limb dominance was determined as their preferred landing leg (Collings, Gorman, Stuelcken, Mellifont, & Sayers, 2020; Ford, Schmitt, Hewett, & Paterno, 2016), of which *n* = 20 were right limb dominant. Previous research has often used the preferred “kicking leg” to determine limb dominance, however, the TJA is primarily a landing assessment, and as such would be more closely related to stability of the stance leg, rather than the kicking action. Following a full outline of the risks and benefits of the procedures, voluntary written consent was obtained. Ethical approval was granted by the institutional research ethics committee (ethics reference: STA-5985) and conformed to the Declaration of Helsinki.

### 2.2. Procedures

Prior to testing, participants performed a standardised 10-min dynamic warm up in the order of: multidirectional jogging, dynamic stretching, bilateral and unilateral jumps, acceleration and deceleration exercises, and change of direction movements. The warm up progressed in intensity of effort, which was consistently observed by the principal investigator. The protocol for the TJA was then described and demonstrated to participants. Participants were instructed to perform continuous maximal height (thighs) tuck jumps for 10-s while adhering to the following instructions: (1) bring the knees up to hip height during each jump, (2) maintain the same landing footprint, and (3) remain facing forward during the test (Myer et al., 2008). Participants were then given an opportunity to familiarize themselves with the test until the principal investigator deemed them technically proficient.

**Fig. 1.**
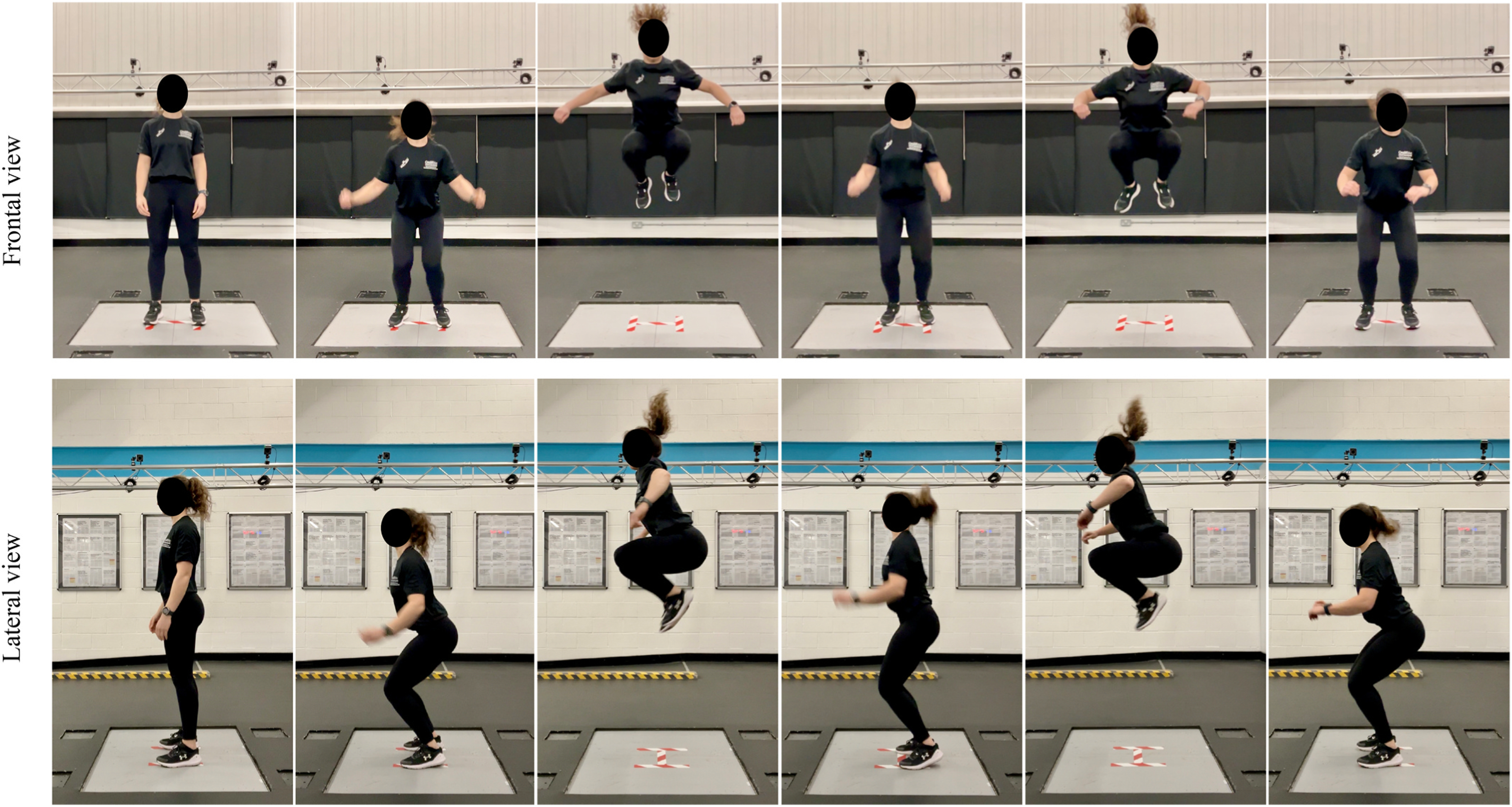
Starting position and example repeated tuck jumps during kinetic assessment of the TJA on dual fixed force plates.

Data acquisition took place atop dual ground-fixed force plates (Type 2812a; Kistler Instruments AG, Winterthur, Switzerland), which were zeroed before each trial of the TJA. Participants were instructed to stand upright with their feet aligned with two vertical strips of tape 35 cm apart, connected with a horizontal line forming an H-shape (Fig. 1). Three successful 10-s trials of the TJA were performed with a 5-min rest period between the 10-s trials. Kinetic data were collected during the TJA trials at a sampling rate of 1000 Hz, with data captured over a 15-s time period to allow for a quiet stance period at the start and end of each trial. Force-time data were instantaneously captured in the manufacturer software (Bioware^®^ Version 5.1) and subsequently exported to Microsoft Excel^®^ and a customised MATLAB^®^ (R2023a, MathWorks Inc., Natick, MA) script. Data were filtered using a recursive, fourth order, low-pass digital Butterworth filter, with a cut-off frequency of 50 Hz determined by residual analysis. The first countermovement jump to initiate the task was excluded from analysis, and the threshold criterion to identify the beginning of each jumping cycle (i.e., initial contact) and the end of the ground contact phase (i.e., take off) was determined when the force exceeded 10 N (Voigt, Dyhre-Poulsen, & Simonsen, 1998).

Force-time data for all trials were normalized to body weight and the trial with the highest mean interlimb asymmetry for peak vertical ground reaction force was then used for statistical analyses. Temporal data for each jumping cycle (i.e., initial contact to take off) within a trial were interpolated to 101 data points (100% of jumping cycle) for SPM waveform analysis. The following discrete kinetic variables were extracted from the bilateral, dominant and non-dominant force-time data: peak VGRF (BW), ground contact time (GCT, s), peak centre of mass displacement (m), relative leg stiffness (*k*_leg_, BW. m^− 1^), average loading rate (BW⋅s^− 1^), instantaneous loading rate (BW⋅s^− 1^), total impulse (BW⋅s), braking impulse (BW⋅s), propulsive impulse (BW⋅s), braking:propulsive impulse ratio, propulsive duration (s), peak braking force (BW), peak propulsive force (BW), time of braking peak (s), and time of propulsive peak (s). Variables were calculated using methods and definitions previously reported (Kember et al., 2022; Pedley, Radnor, Lloyd, & Oliver, 2023) and described in Supplementary Table 1, and all data were reported as mean ± standard deviation. The reliability of kinetics during the TJA have been previously reported, and CV% range from 4.0 to 7.9% for a number of kinetic variables across five successive jumping cycles (Nascimento, Sideris, & Read, 2022).

Stability of kinetics for the bilateral variables was calculated across the jumping cycles for each participant and determined using a sequential averaging technique previously implemented during kinetic assessment of the TJA (Kember et al., 2022; Kember, Myer, Moore, & Lloyd, 2023). Using each variable mean as a criterion measure, stability for each variable was estimated to occur when a cumulative mean, and the cumulative mean of all successive samples, thereafter, were within 0.25 standard deviation of the criterion measure (B. T. Bates, Osternig, Sawhill, & James, 1983; Kember et al., 2022; Racic, Pavic, & Brownjohn, 2009). Interlimb asymmetry for each variable was then calculated for the trials before and after kinetic stabilization. Absolute asymmetry scores between the dominant and non-dominant limbs for all variables were calculated using the Bilateral Asymmetry Index 1: (dominant limb – non-dominant limb)/(dominant limb + non-dominant limb) × 100. This method was used to ensure that interlimb differences measured during the bilateral TJA task were calculated relative to the sum of that variable (Bishop, Read, Chavda, & Turner, 2016; Bishop, Read, Lake, Chavda, & Turner, 2018).

### 2.3. Statistical analyses

Data were examined for normality using a Shapiro-Wilk test, with all data satisfying the criteria of normal distribution (*p* > 0.05). Paired sample t-tests were then performed to determine the differences in bilateral kinetics for all discrete variables, and interlimb kinetic asymmetries for each limb before and after kinetic stabilization. Hedges’ *g* effect sizes were used to determine the magnitude of differences, with effect sizes classified as: trivial (*g* < 0.20) small (*g* = 0.20–0.49), moderate (*g* = 0.50–0.79), and large (*g* = > 0.80) (Cohen, 1988). All statistical analyses were conducted in SPSS^®^ v.27.0 (SPSS Inc., Chicago, IL).

SPM analyses were conducted in MATLAB using open-source software package SPM 1D 0.4 (Pataky, 2012) (v.M0.4, https://spm1d.org/). Specifically, SPM two-tailed paired t-tests were performed to compare the difference between the dominant and non-dominant limbs for all jumping cycles in all trials, and the jumping cycles before and after kinetic stabilization for interlimb analysis (α = 0.05; Hughes-Oliver et al., 2019; Pataky, 2012). SPM analysis involved four steps: (1) computing the value of the test statistic at each point in the normalized time series (i.e., initial contact to take off), (2) estimating temporal smoothness on the basis of the average temporal gradient, (3) computing the critical threshold at which only α = 5% of smooth random curves would be expected to transverse, and (4) computing the probability that specific suprathreshold regions could have resulted from an equivalently smooth random process (Pataky, 2012). Perfect agreement between the dominant and non-dominant limbs would lead to no regions of significant difference, whereas dissimilar waveforms would lead to large regions of difference and thus rejection of the null hypothesis (Pataky, 2010, 2012). Cohen’s *d* effect sizes were used to determine the magnitude of difference between limbs at each time point within the time series and were classified as: trivial (*d* < 0.20) small (*d* = 0.20–0.49), moderate (*d* = 0.50–0.79), and large (*d* = > 0.80) (Cohen, 1988). Positive effect sizes indicate higher values on the dominant limb and negative effect sizes higher on the non-dominant limb.

## 3. Results

Mean kinetics and absolute asymmetries pre- and post-kinetic stabilization are presented in Table 1. Small to moderate increases in asymmetry were observed for VGRF, relative *k*_leg_, average loading rate, total impulse, propulsive impulse, peak braking and propulsive force from pre-to post-kinetic stabilization (*p* < 0.05). Instantaneous loading rate in the non-dominant limb was the only kinetic variable to increase after kinetic stabilization (*p* = 0.02, *g* = 0.48). All other discrete variables and absolute asymmetries showed non-significant, trivial to small changes from pre to post kinetic stabilization (*p* > 0.05). Bilateral data indicated a moderate decrease in peak centre of mass displacement after kinetic stabilization (*p* = 0.01, *g* = −0.54). All other bilateral kinetics showed non-significant, trivial to small differences between stabilization periods (*p* > 0.05). On average, 15 jumping cycles (±1) were performed with stabilization occurring by the 9th jumping cycle (±3) for all discrete variables. Data showed that 56% of the jumping cycles were performed before stabilization and 44% thereafter, suggesting that participants achieved kinetic stability approximately halfway through the 10-s test period.

**Fig. 2.**
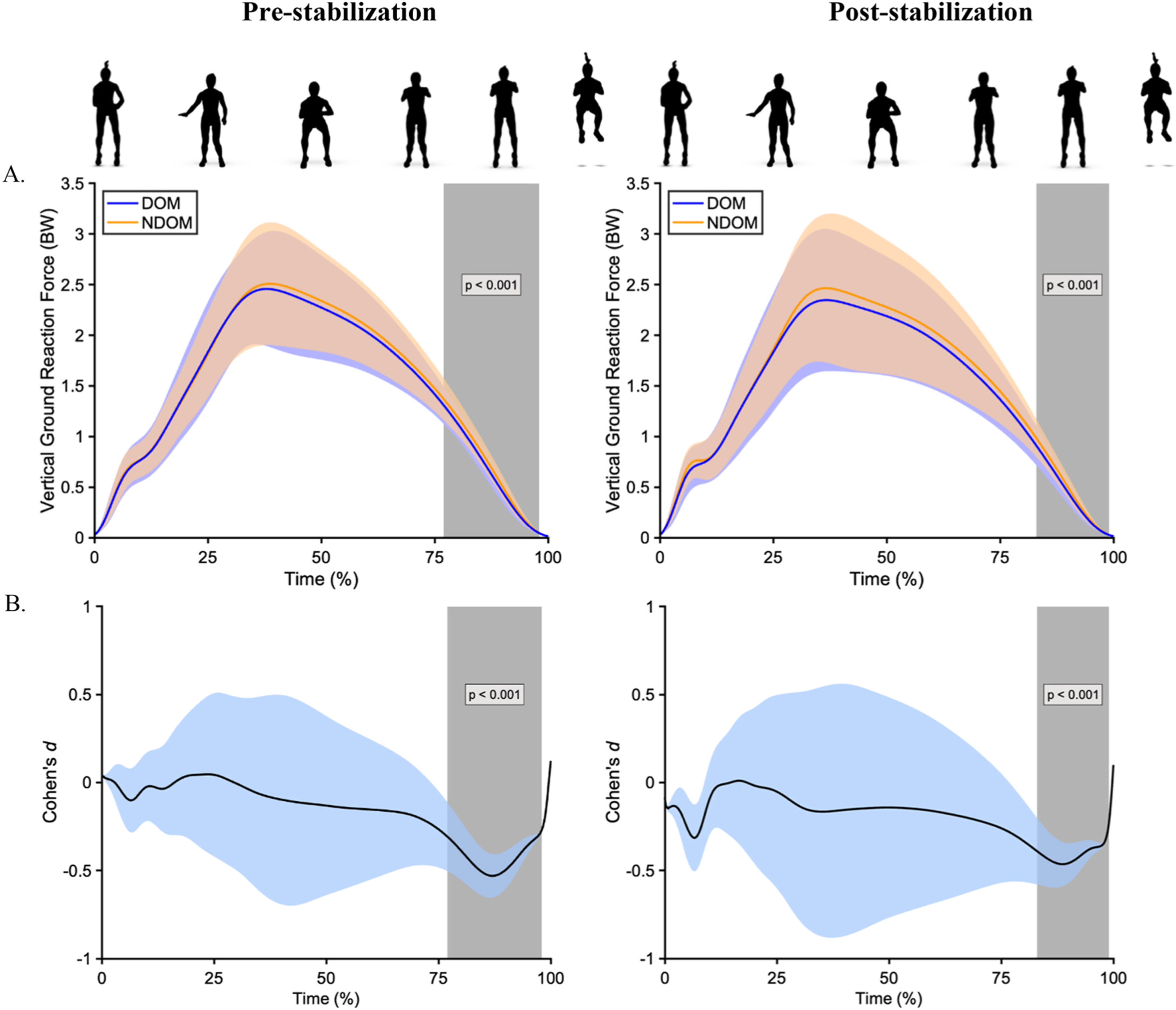
Average TJA vertical ground reaction force waveforms (A) and Cohen’s *d* effect sizes (B) during the jumping cycles pre- and post-stabilization. The grey shaded area represents the supra-threshold cluster that surpassed the critical threshold (t) of 3.08.

The SPM waveform analysis revealed small interlimb differences within one supra-threshold cluster exceeding the critical threshold (t = 3.08) in the region of 77–98% of ground contact during the jumping cycles pre-stabilization (n = 208; *p* < 0.01; *d* = −0.42) and 83–99% of ground contact during the cycles post-stabilization (n = 167; *p* < 0.01; *d* = −0.41) (Fig. 2).

## 4. Discussion

The aims of this study were to (i) examine differences in interlimb kinetic asymmetries of discrete variables during the TJA before and after kinetic stabilization, and (ii) identify differences between limbs before and after kinetic stabilization using discrete time point analysis and SPM waveform analysis. In relation to these aims, our data indicated that absolute asymmetry increased post-kinetic stabilization for various discrete kinetic variables, with increased limb loading evident in the non-dominant limb during the propulsive phase of the jumping cycles pre- and post-stabilization. Consequently, we reject our initial hypothesis that participants would demonstrate greater interlimb asymmetries prior to kinetic stabilization.

The overall magnitude of interlimb kinetic asymmetries presented in this study ranges from 1 to 19%, with 17 participants exceeding the arbitrary 10% threshold for peak VGRF. Asymmetries were notably larger than those previously reported in healthy controls for VGRF, loading rates, and braking and propulsive forces during a drop-jump or countermovement jump (Bakal et al., 2022b; Kotsifaki, Sideris, King, Bahr, & Whiteley, 2023; Read, Auliffe, Wilson, & Graham-Smith, 2020). Interestingly, the absolute asymmetries for these specific variables were also greater in our study when compared to data from the same tasks in patients with ACLR (Bakal et al., 2022b; Katsikari et al., 2020; Read et al., 2020). Absolute asymmetry values presented in this study were more akin to those in younger patients following ACLR performing five-repeated tuck jumps (VanZile et al., 2021). The association between VGRF and knee joint moments indicate that kinetic analysis is a viable method for detecting interlimb compensatory strategies in knee kinetics (Dai, Butler, Garrett, & Queen, 2014). In light of the commonly cited thresholds of 10–15% for interlimb asymmetries, and their suggested relationship to injury risk (Bishop, Turner, & Read, 2018b; Hewit, Cronin, & Hume, 2012), findings in our study indicate that healthy female athletes exhibit interlimb asymmetries that either meet or exceed these suggested thresholds, which could predispose these athletes to an increased risk of ACL injury. However, it is imperative to exercise caution when interpreting injury risk thresholds and findings from our study, as they are based on limited prospective data linking asymmetry variables, specifically during the TJA, to injury incidence. Tracking and monitoring of these asymmetries warrants further exploration but could be considered as a screening tool for practitioners which may enable early identification of athletes who may be at a heightened risk of injury, allowing for timely intervention and preventive measures.

Larger absolute kinetic asymmetries emerged in the jumping cycles following kinetic stabilization, affecting various discrete variables such as VGRF, relative *k*_leg_, average vertical loading rate, propulsive impulse, and peak braking and propulsive force. Notably, emergent asymmetries following kinetic stabilization during the TJA displayed an average 5% increase post kinetic stabilization, surpassing the aforementioned threshold for heightened injury risk. Effect sizes ranged from small to moderate, with more pronounced kinetic changes primarily observed in the non-dominant limb post-stabilization. Despite these asymmetry changes, instantaneous loading rate was the sole kinetic variable that exhibited a significant post-stabilization increase but maintained a similar level of asymmetry both before and after kinetic stabilization. It is worth noting that there is a scarcity of research exploring the interplay of interlimb asymmetries pre- and post-kinetic stabilization. Consequently, while the findings of this study are preliminary, they offer a potentially unique perspective on the strategies employed by female athletes during repetitive tuck jump tasks. In the pursuit of kinetic stability, it appears that participants made a trade-off by sacrificing landing symmetry to meet the technical demands of the task. The observed increase in interlimb asymmetry following kinetic stabilization could thus represent a fundamental landing strategy that prioritizes task completion over movement quality. This rise in asymmetry post-stabilization might also be attributed to acute fatigue experienced in the latter part of the testing period. It is plausible that athletes might be vulnerable to decreased feed-forward responses, which could unveil compensatory strategies stemming from deficiencies in neuromuscular control, strength, and plyometric abilities in one or both limbs, as previously highlighted (Read et al., 2016).

While not the central focus of the study, it is interesting to note that SPM waveform analyses exposed temporally related differences between the dominant and non-dominant limb, with the non-dominant limb exhibiting larger VGRF during the last quarter of ground contact, irrespective of kinetic stabilization. Although the observed difference in magnitude is small, it could be proposed that greater force production in the non-dominant limb may be attributed to, among other factors, a limb dominance strategy or potentially a consequence of reduced neuromuscular adaptations and utilization of the stretch reflex (N. A. Bates, Ford, Myer, & Hewett, 2013). Interlimb variations in propulsive impulse and peak propulsive force, which coincides with this relative phase of ground contact, have been previously reported in healthy athletes and athletes with ACLR (Read et al., 2020). It is well documented that patients with ACLR often redistribute their impulse production to favor the uninvolved limb (Dai et al., 2014). Among the healthy participants included in this study, of which 80% indicated that their right limb was their dominant limb, this phenomenon may also be observable. Results presented in this study could indicate that participants redistributed their impulse production to favor the non-dominant (in this case, left) limb. In order to reduce the incidence of ACL injury, equalisation of side-to-side differences in lower extremity movements, which are emphasised in the TJA, should be considered (Myer et al., 2008). Additional research exploring these temporal differences is important, and practitioners should consider the use of SPM waveform analyses before and after kinetic stabilization when screening athletes for interlimb asymmetries, especially for more “at-risk” populations (e.g., female athletes).

The data presented in this study can serve as a reference point for clinicians and researchers, however it is important to acknowledge certain limitations. Interlimb asymmetries identified in this study are limited to kinetics and it is unclear whether kinematic differences between limbs during the TJA would be apparent, and if these asymmetries would increase in magnitude after kinetic stabilization. Nevertheless, these kinetic asymmetry measures can offer baseline values for healthy female athletes and could be used for comparisons with clinical populations, such as patients who have undergone ACLR. The cross-sectional research design employed in this study limits the ability to establish causal relationships and trends over time. To thoroughly investigate the potential association between TJA kinetic asymmetries and ACL injury risk, future research should prioritize longitudinal and prospective research designs. Research on the interplay of interlimb asymmetries before and after kinetic stabilization remains limited, thereby hindering the ability to draw definitive conclusions. However, the introduction of kinetic stabilization introduces an additional dimension in the assessment of repeated jumping tasks, and warrants further investigation. Furthermore, the study predominantly involved netball athletes, potentially restricting the applicability of the findings to a broader athletic population. To enhance external validity, future studies should incorporate a more diverse participant pool.

## 5. Conclusion

Kinetic analysis of the TJA has revealed an increase in absolute interlimb asymmetry following kinetic stabilization, despite minimal changes to discrete kinetic variables. Interlimb asymmetries post-stabilization often surpassed thresholds commonly associated with injury risk, and warrant further exploration and prospective data. These results highlight the importance of considering interlimb asymmetry and kinetic stabilization in biomechanical assessment of the TJA, and our data offer insights into movement strategies during repeated tuck jumps. From this study, we postulate that athletes may prioritize task completion over landing symmetry, potentially due to fatigue-induced compensatory responses. Furthermore, subtle waveform differences between dominant and non-dominant limbs suggest complexities in limb dominance. When evaluating lower limb biomechanics using the TJA, it seems prudent to consider measuring and monitoring both kinetic stability and interlimb kinetic asymmetries.

## Supplementary Material

Appendix A

## Figures and Tables

**Table 1:** Dominant and non-dominant limb kinetics (mean ± SD) and interlimb asymmetries before and after bilateral kinetic stabilization.

Variable	Stabilization cyde	Before stabilization			After stabilization			Effect Size (*g*)
	
		Dominant	Non-dominant	Absolute asymmetry %	Dominant	Non-dominant	Absolute asymmetry %	
VGRF (BW)	9 ± 4	2.62 ± 0.41	2.63 ± 0.43	9.42	2.57 ± 0.54	2.63 ± 0.52	**15.71** ^a^	−0.60
GCT (s)	9 ± 3	0.24 ± 0.09	0.24 ± 0.09	2.55	0.23 ± 0.04	0.23 ± 0.03	2.40	0.11
*k*_leg_ (kN.m^−1^)	8 ± 4	17.19 ± 3.97	17.40 ± 4.11	9.26	18.00 ± 5.32	18.35 ± 4.72	**14.80** ^a^	−0.51
LR_Av_ (BW⋅s^−1^)	9 ± 4	55.55 ± 12.10	54.86 ± 12.80	13.20	54.59 ± 16.97	54.64 ± 14.29	**18.70** ^a^	−0.45
LR_Ins_ (BW⋅s^−1^)	8 ± 3	70.59 ± 19.03	69.06 ± 14.95	14.90	76.42 ± 17.14	**76.48 ± 13.70** ^b^	15.84	−0.10
Imp_Total_ (BW⋅s)	9 ± 3	0.33 ± 0.08	0.33 ± 0.07	8.35	0.32 ± 0.05	0.32 ± 0.04	**13.02** ^a^	−0.68
Brake_Imp_ (BW⋅s)	8 ± 4	0.19 ± 0.11	0.19 ± 0.12	9.96	0.16 ± 0.03	0.16 ± 0.02	13.13	−0.35
Prop_Imp_ (BW⋅s)	9 ± 4	0.18 ± 0.09	0.17 ± 0.09	10.74	0.16 ± 0.03	0.16 ± 0.02	**14.67** ^a^	−0.40
Brake: Prop	9 ± 3	1.05 ± 0.11	1.02 ± 0.07	8.33	1.06 ± 0.12	1.01 ± 0.10	7.53	0.18
Prop_Imp_ time (s)	9 ± 4	0.14 ± 0.09	0.13 ± 0.09	5.23	0.12 ± 0.03	0.12 ± 0.02	4.44	0.24
Brake_Peak_ (BW)	9 ± 3	2.62 ± 0.41	2.62 ± 0.43	9.70	2.57 ± 0.53	2.63 ± 0.52	**15.57** ^a^	−0.55
Prop_peak_ (BW)	9 ± 3	2.28 ± 0.42	2.32 ± 0.44	7.66	2.28 ± 0.48	2.34 ± 0.50	**12.88** ^a^	−0.56
Brake_peak_ time (s)	9 ± 3	0.08 ± 0.01	0.08 ± 0.01	6.08	0.08 ± 0.01	0.08 ± 0.01	6.59	−0.12
Prop_peak_ time (s)	9 ± 3	0.13 ± 0.09	0.13 ± 0.09	1.32	0.11 ± 0.02	0.11 ± 0.02	1.11	0.12

VGRF – peak vertical ground reaction force; GCT – ground contact time; k_leg_ – leg stiffness; LR_Av_ – average loading rate; LR_Ins_ – instantaneous loading rate; Imp_Total_ – total impulse; Brake_Imp_ – braking impulse; Prop_Imp_ – propulsive impulse; Brake:Prop – braking:propulsive phase ratio; Brake_Peak_ – peak braking force; Prop_Peak_ – peak propulsive force; BW – body weight; g – Hedge’s g effect size.

a significant difference in asymmetries p < 0.05.

b significant difference in kinetics of the same limb p < 0.05.
