# Supplementary material for: Interlimb kinetic asymmetries during the tuck jump assessment are more exposed following kinetic stabilization
Source: Phys Ther Sport. Author manuscript; Available in PMC 2025 May 1. (PMC11178106; doi:10.1016/j.ptsp.2024.03.002)
Supplement: Appendix A [file NIHMS1993729-supplement-Appendix_A.docx]

| **Supplementary Table 1. Kinetic variable definitions** | |
| --- | --- |
| **Variable** | **Description** |
| Peak vertical ground reaction force (VGRF) [BW] | Peak vertical ground reaction force during the active peak of each jumping cycle. |
| Ground contact time (GCT) [s] | Time between each touch-down and take-off. |
| Peak centre of mass displacement [m] | Peak vertical displacement of the body’s centre of mass during ground contact was determined by dividing the vertical ground reaction force by body mass to determine acceleration. Centre of mass displacement was then calculated through the double integration of acceleration from initial ground contact. |
| Vertical relative leg stiffness (*k*_leg_) [BW·m^–1^] | Ratio of peak vertical ground reaction force to the corresponding centre of mass displacement at the time of peak vertical ground reaction force ^1^. |
| Vertical average loading rate (VALR) [BW·s^–1^] | The average change in force between 20 and 80% of the time to peak impact ^3^. |
| Vertical instantaneous loading rate (VILR) [BW·s^–1^] | The maximum change in force between each time point within 20 and 80% of the time to peak impact. |
| Total impulse (Imp_Total_ ) [BW·s] | The integral of the vertical ground reaction force using a trapezoidal rule ^2^ for the duration of ground contact. |
| Braking impulse (Brake_Imp_) [BW·s] | The total vertical impulse applied to the ground during the braking phase of ground contact. It is calculated by summing the instantaneous impulse values between initial ground contact and peak center of mass displacement ^4^. |
| Proulsive impulse (Prop_Imp_) [BW·s] | The total vertical impulse applied to the ground during the propulsive phase of ground contact. It is calculated by summing the instantaneous impulse values between peak center of mass displacement and take-off ^4^. |
| Braking:propulsive impulse ratio (Brake:Prop) | The ratio of braking impulse and propulsive impulse. |
| Propulsive duration (Prop_Imp_ time)[s] | The time interval between peak center of mass displacement and take-off. |
| Peak braking force (Brake_Peak_)[BW] | The force transient with the greatest magnitude during the braking phase. |
| Peak propulsive force (Prop_Peak_) [BW] | The force transient identiﬁed between peak center of mass displacement and take-off. |
| Time of braking peak (Brake_Peak_ time) [s] | Time of peak braking force from initial ground contact. |
| Time of propulsive peak (Prop_Peak_ time) [s] | Time of peak propulsive force from initial ground contact. |

1. Brazier J, Maloney S, Bishop C, Read PJ, Turner AN. Lower Extremity Stiffness: Considerations for Testing, Performance Enhancement, and Injury Risk. *J Strength Cond Res*. 2019;33(4):1156-1166. doi:10.1519/JSC.0000000000002283

2. Enoka RM. *Neuromechanics of Human Movement*. 4th ed. Human Kinetics; 2008.

3. Milner CE, Ferber R, Pollard CD, Hamill J, Davis IS. Biomechanical factors associated with tibial stress fracture in female runners. *Med Sci Sports Exerc*. 2006;38(2):323-328. doi:10.1249/01.mss.0000183477.75808.92

4. Pedley JS, Radnor JM, Lloyd RS, Oliver JL. Analyzing Drop Jump Ground Reaction Forces in Microsoft Excel. *Strength Cond J*. Published online April 12, 2023. doi:10.1519/SSC.0000000000000776
